# Boarding Duration in the Emergency Department and Inpatient Delirium and Severe Agitation

**DOI:** 10.1001/jamanetworkopen.2024.16343

**Published:** 2024-06-11

**Authors:** Joshua W. Joseph, Noémie Elhadad, Melissa L.P. Mattison, Lauren M. Nentwich, Sharon A. Levine, Edward R. Marcantonio, Maura Kennedy

**Affiliations:** 1 Department of Emergency Medicine, Brigham and Women’s Hospital, Boston, Massachusetts; 2 Department of Emergency Medicine, Harvard Medical School, Boston, Massachusetts; 3 Department of Computer Science, Columbia University, New York, New York; 4 Department of Biomedical Informatics, Columbia University, New York, New York; 5 Department of Medicine, Massachusetts General Hospital, Boston; 6 Department of Medicine, Harvard Medical School, Boston, Massachusetts; 7 Department of Emergency Medicine, Massachusetts General Hospital, Boston; 8 Department of Medicine, Beth Israel Deaconess Medical Center, Boston, Massachusetts

## Abstract

This cohort study examines the risk factors for developing delirium and/or agitation in patients in the emergency department waiting for an inpatient bed.

## Introduction

Recent studies have demonstrated that emergency department (ED) boarding (ie, holding admitted patients in the ED while awaiting an inpatient bed) is associated with higher mortality in older adults and that longer lengths of stay and hallway time are associated with developing delirium.^1,2,3^ With rising rates of ED boarding,^4^ understanding the adverse outcomes of boarding and people most likely to be harmed is important. This study aimed to evaluate the association of ED boarding duration with inpatient delirium and agitation.

## Methods

In this retrospective cohort study, we examined visits of adult patients to Mass General Brigham EDs between January 1, 2018, and December 31, 2022, who were subsequently admitted to general medicine services. Patients admitted with primary psychiatric diagnoses or to intensive care units were excluded. We included EDs at 2 academic and 5 community hospitals, with 15 000 to 117 760 visits yearly. The Mass General Brigham Institutional Review Board deemed this study exempt from review and waived the informed consent requirement because retrospective anonymized data were used. We followed the STROBE reporting guideline.

The composite outcome was presence of delirium or severe agitation during admission as defined by an *International Statistical Classification of Diseases and Related Health Problems, Tenth Revision* (*ICD-10*) code for delirium, a nursing screen positive for delirium, use of parenteral antipsychotics, and/or use of physical restraints. Boarding duration was defined as the time interval between 2 hours after inpatient admission request and ED departure for signout and transport. We identified patients with dementia by *ICD-10* codes (eMethods in Supplement 1).

Population differences were evaluated using independent, 2-tailed *t* tests and Fisher Exact tests, with overall significance of *P* < .01. Logistic regression was used to evaluate the association of boarding time in hours with the composite outcome, adjusting for age, dementia, and interaction terms. Analysis was performed using SciPy in Python (Python Software Foundation). All interaction terms were evaluated; we report the final parsimonious model via Akaike information criterion.

## Results

A total of 236 169 patients (121 306 females [51.4%], 114 863 males [48.6%]; mean age, 66.5 [18.2] years) were included (Table). Across all patients, mean ED boarding time was 174.8 (95% CI, 174.1-175.4) minutes.

**Table.** Characteristics of the Study Population and Association of Boarding Time With Inpatient Delirium and/or Agitation^a^

Characteristic	Patients, No. (%)			*P* value
	Total	Without delirium or agitation	With delirium or agitation	
Patients	236 169 (100)	219 902 (88.5)	27 194 (11.5)	
Age, mean (95% CI), y	66.5 (66.4-66.6)	65.7 (65.6-65.7)	72.9 (72.7-73.1)	<.001
Sex				
Male	114 863 (48.6)	101 195 (48.4)	13 526 (50.3)	.02
Female	121 306 (51.4)	107 780 (51.6)	13 668 (49.7)	
Preexisting dementia	4716 (2.0)	3513 (1.7)	1203 (4.4)	<.001
ED boarding time, mean (95% CI), min	174.8 (174.1-175.5)	172.4 (171.7-173.1)	206.9 (203.8-210.1)	<.001
Patients boarding <4 h	221 398 (93.7)	196 578 (94.1)	24 820 (91.3)	<.001
Patients boarding 4-8 h	9290 (3.9)	7.837 (3.6)	1438 (5.3)	<.001
Patients boarding >8 h	5479 (2.3)	4558 (2.2)	921 (3.4)	<.001

Abbreviation: ED, emergency department.

^a^ Boarding time is defined as the time between placement of bed admission request and departure (for signout or transport) from the ED.

Patients who experienced delirium or agitation (27 194 [11.5%]) vs those without delirium or agitation (219 902 [88.5%]) were older (72.9 years vs 65.7 years; *P* < .001) and more likely to have dementia (1203 [4.4%] vs 3513 [1.7%]; *P* < .001). They also had longer mean ED boarding duration (206.9 [95% CI, 203.8-210.1] minutes vs 172.4 [95% CI, 171.7-173.1] minutes; *P* < .001).

Logistic regression demonstrated odds of association given preexisting dementia (odds ratio [OR], 2.04; 95% CI, 1.90-2.19), increasing age in years (OR, 1.07; 95% CI, 1.05-1.09), and each hour of boarding (OR, 1.02; 95% CI, 1.02-1.02) as well as a small interaction between boarding duration and dementia (OR, 1.00; 95% CI, 1.00-1.00). Compared with a 50-year-old patient boarding for 2 hours, a 75-year-old patient boarding for 4 hours has over double the risk of developing delirium. With a preexisting diagnosis of dementia and boarding for 8 hours, this risk increases more than 7-fold.

## Discussion

The findings suggest that ED boarding duration is a direct risk factor for developing delirium or severe agitation during inpatient admission. Building on prior study results,^1,2,3^ we found this risk to be magnified in patients with dementia.

Study limitations include potential underdiagnosis of both dementia and delirium, which could dilute associations; use of a composite outcome; and potential for delirium to complicate bed placement, prolonging boarding. The findings support a growing body of literature that associates prolonged ED stays with substantial harms in older patients.^1,2,3^ Strategies to mitigate this risk should be developed and targeted to patients aged 65 years or older, particularly those with dementia. Given the association of delirium with longer inpatient lengths of stay,^5,6^ risk mitigation may increase inpatient bed availability, a major factor in ED crowding.

## Notes

Supplement 1.
eMethods.

Supplement 2.
Data Sharing Statement
