# Supplementary material for: Boarding Duration in the Emergency Department and Inpatient Delirium and Severe Agitation
Source: JAMA Netw Open. 2024 Jun 11;7(6):e2416343. doi: 10.1001/jamanetworkopen.2024.16343 (PMC11167494; doi:10.1001/jamanetworkopen.2024.16343)
Supplement: Supplement 1. — eMethods. [file jamanetwopen-e2416343-s001.pdf]

Supplemental Online Content

Joseph JW, Elhadad N, Mattison ML, et al. Boarding duration in the emergency department and inpatient delirium and severe agitation. *JAMA Netw Open*. 2024;7(6):e2416343. doi:10.1001/jamanetworkopen.2024.16343

eMethods.

This supplemental material has been provided by the authors to give readers additional information about their work.

© 2024 Joseph JW et al. *JAMA Network Open*

eMethods.

Inclusion & Exclusion Criteria:

ICD10 Codes Used for Prior Dementia Diagnosis

ICD10 Code	Diagnosis
A81.00	Creutzfeldt-Jakob disease, unspecified
B20, F02.80	AIDS dementia complex without behavioral disturbance
B20, F02.81	AIDS dementia complex with behavioral disturbance
F01	Vascular Dementia
F01.50	Vascular dementia without behavioral disturbance
F01.51	Vascular dementia with behavioral disturbance
F02.80	Dementia in other diseases classified elsewhere without behavioral disturbance
F02.81	Dementia in other diseases classified elsewhere with behavioral disturbance
F03.90	Unspecified dementia without behavioral disturbance
F03.90	Nonorganic dementia without behavioral disturbance
F03.91	Unspecified dementia with behavioral disturbance
F03.91	Nonorganic dementia with behavioral disturbance
F07.81	Boxer's Dementia
F10.27	Alcohol dependence with alcohol-induced persisting dementia
F10.97	Alcohol use, unspecified with alcohol-induced persisting dementia
G10	Huntington's disease
G20, F02.80	Parkinson's dementia without behavioral disturbance
G20, FO2.81	Parkinson's dementia with behavioral disturbance
G30.0	Alzheimer's disease with early onset
G30.0, F02.80	Alzheimer's disease with early onset without behavioral disturbance
G30.0, F02.81	Alzheimer's disease with early onset with behavioral disturbance
G30.1	Alzheimer's disease with late onset
G30.1, F02.80	Alzheimer's disease with late onset without behavioral disturbance
G30.1, F02.81	Alzheimer's disease with late onset with behavioral disturbance
G30.8	Other Alzheimer's disease
G30.9	Alzheimer's disease, unspecified
G31.01	Pick's disease
G31.09	Other frontotemporal dementia
G31.1	Senile degeneration of brain, not elsewhere classified
G31.1	Senile degeneration of brain, not elsewhere classified

G31.2	Degeneration of nervous system due to alcohol
G31.81	Alpers disease
G31.83	Dementia with Lewy bodies
G31.84	Mild cognitive impairment, so stated
G31.85	Corticobasal degeneration
G31.9	Degenerative disease of nervous system, unspecified
G91.2	(Idiopathic) normal pressure hydrocephalus
I67.3	Progressive vascular leukoencephalopathy
I69.31	Cognitive deficits following cerebral infarction
I69.31	Cognitive deficits following cerebral infarction
I69.81	Cognitive deficits following other cerebrovascular disease
I69.81	Cognitive deficits following other cerebrovascular disease
I69.91	Cognitive deficits following unspecified cerebrovascular disease
I69.91	Cognitive deficits following unspecified cerebrovascular disease
R41.0	Delirium with Dementia
Z86.59	History of Dementia

Psychiatric Diagnosis Exclusions

Hospitalization for primary psychiatric diagnosis
Legal order for emergency restraint and hospitalization of persons posing risk of serious harm by reason of mental illness (Section 12 order)
Transfer to psychiatric care facility
Psychiatric restraint order at any time during ED/Inpatient Stay

Covariate and Outcome Definitions

ICD10 Codes Used for Delirium Diagnosis

290.11	Presenile dementia with delirium
290.3	Senile dementia with delirium
290.41	Vascular dementia, with delirium
293.0	Delirium due to conditions classified elsewhere
293.1	Subacute delirium
780.09	Alteration of consciousness other

© 2024 Joseph JW et al. *JAMA Network Open*

F05	Delirium due to known physiological condition
R41.0	Disorientation, unspecified
G92	Toxic encephalopathy
G93.4X	Other and unspecified encephalopathy

Parenteral Antipsychotic Administration:
Defined as administration of any medication within the medication class “Antipsychotic” and administered parenterally (intramuscular or intravenous administration).

Positive Nursing Delirium Screen:
Defined as a positive CAM-ICU score. (Ely EW, Margolin R, et al. Evaluation of delirium in critically ill patients: validation of the Confusion Assessment Method for the Intensive Care Unit (CAM-ICU). Crit Care Med. 2001 Jul;29(7):1370-9.)

© 2024 Joseph JW et al. *JAMA Network Open*
