# Supplementary material for: Boarding Duration in the Emergency Department and Inpatient Delirium and Severe Agitation
Source: JAMA Netw Open. 2024 Jun 11;7(6):e2416343. doi: 10.1001/jamanetworkopen.2024.16343 (PMC11167494; doi:10.1001/jamanetworkopen.2024.16343)
Supplement: Supplement 2. — Data Sharing Statement [file jamanetwopen-e2416343-s002.pdf]

Data Sharing Statement

Joseph. Boarding Duration in the Emergency Department and Inpatient Delirium and Severe Agitation. *JAMA Netw Open*. Published June 11, 2024.
doi:10.1001/jamanetworkopen.2024.16343

Data
Data available: No
